# Women with PCOS have a heightened risk of cardiometabolic and cardiovascular diseases: statement from the Experts Group on Inositol in Basic and Clinical Research and PCOS (EGOI-PCOS) and Italian Association of Hospital Cardiologists (ANMCO)

**DOI:** 10.3389/fcvm.2025.1520490

**Published:** 2025-09-02

**Authors:** Giovanna Geraci, Carmine Riccio, Fabrizio Oliva, Domenico Gabrielli, Furio Colivicchi, Massimo Grimaldi, Fabio Facchinetti, Vittorio Unfer

**Affiliations:** ^1^ UOC Cardiology-UTIC and Hemodynamics, Sant’Antonio Abate, Erice, Italy; ^2^ Cardiovascular Department, AORN Sant’Anna e Sant’Sebastiano di Caserta, Caserta, Italy; ^3^ Cardiology 1-Hemodynamics, Cardio-Thoraco-Vascular Department “A. De Gasperis”, ASST Grande Metropolitano Niguarda Hospital, Milan, Italy; ^4^ ANMCO—Italian Association of Hospital Cardiologists, Florence, Italy; ^5^ Foundation for Your Heart, Florence, Italy; ^6^ UOC Cardiology, Cardio-Thoraco-Vascular Department, A.O. San Camillo Forlanini, Rome, Italy; ^7^ UOC Clinical and Riabilitative Cardiology, San Filippo Neri Hospital, ASL Roma 1, Rome, Italy; ^8^ UOC Cardiology-UTIC, Miulli Hospital, Acquaviva Delle Fonti, Italy; ^9^ Mother-Infant Department, University of Modena and Reggio Emilia, Modena, Italy; ^10^ The Experts Group on Inositol in Basic and Clinical Research and on PCOS (EGOI-PCOS), Rome, Italy; ^11^ Department of Gynecology and Obstetrics, UniCamillus-Saint Camillus International University of Health Sciences, Rome, Italy

**Keywords:** cardiovascular diseases, PCOS, CVD prevention, CVD risk factors, OCPs

## Abstract

In recent years, the prevalence of polycystic ovary syndrome (PCOS) has gradually increased, and the investigation of the causal factors influencing etiopathogenesis is attracting attention. Several studies have highlighted that patients with PCOS exhibit an increased risk of cardiovascular disease (CVD) compared with healthy people, and these risks include the occurrence of myocardial infarction, ischemic heart disease, and stroke. This correlation becomes particularly important when PCOS is diagnosed and consequently a specific treatment is recommended. Of note, women with PCOS may exhibit different pathological features even if quite often they are considered as a sole unique group of patients. Interestingly, the rate of CVD occurrence is differently linked to PCOS phenotypes. Data from literature demonstrate that CVD risks are strongly associated with those comorbidities frequently observed in PCOS, mainly metabolic alterations such as hypertension, abdominal obesity, dyslipidemia, insulin resistance, and atherosclerosis, that predispose individuals to CVD and type 2 diabetes. Women presenting PCOS, particularly those with a hyperandrogenic pattern, seem more prone to develop CVD with respect to other PCOS patients. This may be related to genetic factors, dyslipidemia, and hypertension combined with excessive androgen, which may explain the increased risk factor of CVD in patients with PCOS. On these premises, it becomes important to implement the therapeutic rationale and the risk assessment before treatment prescription and to encourage meticulous patient observation during medical examinations. This aspect becomes crucial particularly in adolescent patients, as in many cases, PCOS may be predictive of CVD occurrence.

## 1 Introduction

Cardiometabolic risk has been identified as a cluster of metabolic and cardiovascular abnormalities, including hypertension, abdominal obesity, insulin resistance (IR), dyslipidemia, and atherosclerosis, that predispose individuals to cardiovascular disease (CVD) and type 2 diabetes (T2D). CVD, T2D, and overweight/obesity are extremely interrelated conditions. For example, approximately 70% of total mortality in T2D is due to CVD, and individuals with metabolic syndrome are at increased risk of developing T2D and CVD (1). The prevalence and impact of these cardiometabolic diseases (CMD) are enormous. The World Health Organization estimates that in 2022, 2.5 billion adults (18 years and older) were overweight. Of these, 890 million were living with obesity (2), and 17.9 million people died from CVDs in 2019, representing 32% of all global deaths (3). In addition to increased mortality risk, CVD, diabetes, and obesity are associated with reduced quality of life and an increased economic burden on both the individual and society. Thus, an important goal of global public health is to reduce cardiometabolic risk.

An increasing amount of evidence suggests an association of CVDs with polycystic ovary syndrome (PCOS), as patients with this pathology frequently carry various comorbidities that may increase the risk of developing CVD (4). Indeed, a huge percentage of women with PCOS also exhibit cardiovascular risk factors (CVRFs), including dyslipidemia, IR, hypertension, hypertensive disorders in pregnancy, gestational and early-onset diabetes, and metabolic syndrome (5). Compared with the general population, these CVRFs often manifest at an earlier age in women with PCOS, a population where the prevalence of excess body weight is also particularly high (6).

To date, the huge scientific interest in PCOS is also characterized by difficulties in reaching an alignment between the various scientific societies to achieve a common diagnostic process. These divergences translate into excessive variability in the diagnosis of women with PCOS and into an overlooking of CVD risk characterizing these patients since adolescence. In the present manuscript, we focused on the important connection between PCOS and CVD, underlying the genetic traits that may be shared by these conditions and describing how to choose the clinical approach starting from adolescents presenting with both PCOS and CVRFs.

## 2 Definition and main features of CVDs

For requirements for a specific article type, CVDs are defined as a group of disorders of the heart and blood vessels. They include atherosclerotic diseases (affecting coronary, cerebrovascular, and peripheral circulation), rheumatic heart disease (caused by streptococcal bacteria), congenital heart disease, deep vein thrombosis, and pulmonary embolism (7).

Heart attacks and strokes are also included in the definition of CVD and consist of acute events mainly caused by an obstruction of the blood vessels that reach the heart or brain. They are commonly derived by the formation of atherosclerotic plaques (fatty deposits on the inner walls of the blood vessels) with subsequent blood clot layering and vessel blockage which may lead to ischemia of cerebral or myocardial tissue. Strokes can also be caused by bleeding from a blood vessel in the brain (3).

A “risk factor” is a variable that increases the chances of developing a disease. In many cases, a single risk factor may be linked to one or more CVDs (8). In some cases, patients can present cardiometabolic risk (CMR) which refers to risk factors that increase the possibility of vascular issues or diabetes onset. This concept encompasses traditional risk factors included in risk calculators, such as hypertension, dyslipidemia, and smoking, as well as emerging risk factors, such as abdominal obesity, inflammatory profile, and ethnicity (9). The centrality of the metabolic syndrome is well established, but since it cannot itself include global CVD risk, the term cardiometabolic risk was introduced to fill that gap. CMR identifies the overall CVD risk associated with the canonical risk factors to which we add an additional risk derived from the features of metabolic syndrome.

It can be stated that some recognized risk factors for CVD are well documented to date and include age, sex, family history, dyslipidemia, hypertension, dysglycemia, and smoking. Moreover, there are newer cardiovascular risk factors that are added to the traditional ones, such as IR, abdominal obesity (related to waist circumference), increased inflammation status revealed as high-sensitivity C-reactive protein (hsCRP) levels, lack of consumption of fruits and vegetables, sedentary lifestyle, and psychosocial stress. In several patients with elevated serum levels of triglycerides, it could be useful to measure the quantity of apolipoprotein B in place of low-density lipoprotein cholesterol (LDL-C) to better assess the CMR (10). It seems increasingly important to include the evaluation of those new parameters in the routine analysis, given that the early identification of risk factors for medical conditions is one of the main objectives in the field of epidemiology. Indeed, most of these factors should be addressed by primary care providers (11).

## 3 PCOS patients are exposed to increased CVD risk compared with healthy controls

PCOS is a complex disorder presented by women of reproductive age, with a prevalence estimated from 8% to 13% worldwide (12). Women with PCOS also commonly present with infertility, complications of pregnancy, obesity, IR, hirsutism, acne, androgenic alopecia, and mood disorders (13).

Several data sustain a more pronounced predisposition of female individuals to develop CVD complications when PCOS is diagnosed. Hence, women with PCOS have long-term exposure to traditional CVRFs as they often present with obesity, IR, abnormal glucose metabolism, dyslipidemia, and elevated blood pressure as early as young adulthood or even adolescence (14). Also, a recent meta-analysis observed that normal weight children of women with PCOS were more exposed to CVD and CMD risks in early childhood (15).

An important systematic review and meta-analysis was recently published in the *Journal of the American Heart Association*, which describes a comparison of the risk of clinical CVD events in women with and without PCOS (16). The results of the investigation indicate that PCOS correlates with higher risk of composite CVD [OR, 1.68 (95% CI, 1.26–2.23); *I*^2^ = 71.0%], composite ischemic heart disease [OR, 1.48 (95% CI, 1.07–2.05); *I*^2^ = 81.0%], myocardial infarction [OR, 2.50 (95% CI, 1.43–4.38); *I*^2^ = 83.3%], and stroke [OR, 1.71 (95% CI, 1.20–2.44); *I*^2^ = 81.4%].

The investigation regarding the correlation between PCOS and the high prevalence of CVDs has also been described in preclinical studies. Indeed, Mannerås et al. (17) developed a letrozole-induced rat model of PCOS that exhibits both ovarian and metabolic features of the syndrome. These animals showed insulin resistance, dyslipidemia, and elevated blood pressure, all of which are commonly recognized as risk factors for CVDs. Similarly, Kauffman et al. (18) demonstrated that hyperandrogenic female rats presented with endothelial dysfunction and increased arterial stiffness, further supporting the vascular consequences of excess androgens. These preclinical models mirror the human phenotype and provide mechanistic insights into the pathophysiological pathways, particularly the role of hyperinsulinemia and androgen excess, in promoting cardiovascular alterations.

Data regarding the strong association of PCOS with CVD is constantly increasing, and evidence has also been collected with novel approaches. As an example, a cross-sectional study used a large US-based digital cohort of iPhone users enrolled between 2019 and 2022, with data monitored through an Apple Research application (19). In this study, 60,789 participants were included, and the results strongly highlight that PCOS was significantly associated with a higher prevalence of all metabolic and several cardiovascular conditions. Indeed, women with PCOS reported higher arrhythmia (POR, 1.37; 95% CI, 1.20–1.55), coronary artery disease (POR, 2.92; 95% CI, 1.95–4.29), heart attack (POR, 1.79; 95% CI, 1.23–2.54), and stroke (POR, 1.66; 95% CI, 1.21–2.24). Interestingly also the metabolic alteration were more pronounced in PCOS group, including obesity (POR, 2.94; 95% CI, 2.77–3.12), prediabetes (POR, 3.75; 95% CI, 3.47–4.06), type 1 diabetes (POR, 1.43; 95% CI, 1.07–1.90), type 2 diabetes (POR, 2.76; 95% CI, 2.43–3.15), high cholesterol (POR, 1.68; 95% CI, 1.55–1.81), hypertension (POR, 1.57; 95% CI, 1.45–1.70), and metabolic syndrome (POR, 3.28; 95% CI, 2.94–3.66). The same observations were found in those women who declared a prolonged time to reach the regularity of the menstrual cycle.

The clinical management for women presenting with PCOS quite often regards infertility issues, menstrual cycle irregularity, and clinical signs of hyperandrogenism as hirsutism or acne. However, even if these patients have increased CVD risk as well as increased prevalence of dysmetabolism, the screening for CVRFs is frequently underestimated and neglected in clinical practice.

## 4 Ethnicity, age, and specific biomarkers to improve the diagnosis

PCOS presents an elevated metabolic and clinical heterogeneity among different ethnic groups, thus influencing both the diagnosis and the CVD risk associated. These variations derive from different genetic, environmental, and socioeconomic factors characterizing each population. Indeed, an analysis of data from the Dallas Heart Study found that women with PCOS had a higher prevalence of hypertension, hypercholesterolemia, hypertriglyceridemia, and impaired fasting glucose than controls, with significant variations across ethnic groups (20).

An example of how the clinical manifestation of PCOS may differ among the population was recently reported by Joham et al. (21). They observed that Asiatic and American women with PCOS were more frequently characterized by a metabolic phenotype with central obesity, IR, and an increased risk for T2D regardless of BMI, while European and Middle Eastern women with PCOS exhibit an hyperandrogenic trend with a pronounced hirsutism and androgenetic alopecia.

A comparative study on PCOS compared Middle Eastern and UK women, showing that even though both cohorts were at major CVD risk compared with the healthy population, they differ in the specific characteristics of each group (22). The British women present with elevated BMI, blood pressure, and triglycerides, while the Middle Eastern group had increased levels of testosterone, HDL, and C-reactive protein (CRP).

Interestingly, this variability also reflects a different CVD risk between different ethnic groups (23). Indeed, black women exhibit an increased trend for elevated blood pressure and a greater CVD risk even with a favorable lipidic profile. The Hispanic group showed a greater tendency for obesity and diabetes, with a less favorable lipid profile. The PCOS from the Asiatic group had a reduced rate of obesity, but they present with an increased prevalence of dyslipidemia and IR.

The variability in PCOS diagnosis and associated CVRFs should not be considered as globally valid since data suggest a differentiation according to ethnicity.

Moreover, PCOS also exhibits a great variability in CVD risk during different periods of a human’s life. Different age reflects physiological variations due to hormonal and metabolic changes typical of an individual’s maturation. In young women with PCOS (15–30 years), CVRFs present an increased rate with respect to the controls. Particularly, the prevalence of PCOS among pubertal patients with overweight or obesity is approximately 22%, which is notably higher than that observed in adult patients (24). In addition, studies focusing on adolescents already diagnosed with PCOS have shown that 21%–50% of these individuals were overweight or obese, with a waist circumference five times greater than that of the control group (25–27). These patients exhibit an elevated risk of developing CVDs during aging.

In the age range 30–40 years, women with PCOS have a 19% higher risk of developing CVDs, and this can be attributed to various factors including obesity, hypertension, and T2D which seem more prevalent in young women (28). Even in the absence of obesity or dyslipidemia, young women with PCOS are more prone to exhibit precocious features of endothelial dysfunction and carotid intima–media thickening, which feature arteriosclerosis (29). These patients exhibit a significant difference in flow-mediated dilation and intima–media compared with controls. Also, the levels of serum endothelin-1 are significantly higher in this population of PCOS patients (30). Moreover, the association of hyperandrogenism and insulin resistance frequently observed at a very young age tends to worsen with increasing body weight. Specifically, there is a progressive decline in endothelial function from lean to overweight and obese women with PCOS. Excess visceral fat accumulation is also a significant predictor of atherosclerosis in this population (29).

In middle-aged women (35–55 years), some PCOS features as hyperandrogenism, and oligo-/anovulation seems to decrease with aging. However, quite often women with PCOS can still exhibit an adverse cardiometabolic profile. A study demonstrates that 50-year-old women with PCOS showed a significant increase in waist circumference, BMI, and blood pressure compared with healthy controls even if the lipid profile resulted similar (31).

A recent study demonstrates that PCOS women have a hazard ratio of 2.33 with respect to controls when diagnosis is performed according to Rotterdam criteria and a hazard ratio of 2.47 with respect to controls when diagnosis is performed according to NIH criteria. Interestingly, the authors observe a divergence in the whole curve of cumulative hazard starting from the age of 35 (32).

Eventually, in menopausal women (>55 years) with PCOS, CVDs are less noticeable even if CMDs are more present with respect to their healthy counterparts. Nevertheless, the adverse cardiovascular events are less frequent with aging, even if the presence of hyperandrogenism and other canonical PCOS features still exposes these postmenopausal women with PCOS to an increased risk of CVDs compared with controls (33). In detail, a “dose–response” relationship was observed: women with more PCOS traits (e.g., hyperandrogenism and menstrual irregularity) had increased odds of CVD. Specifically, those with all three PCOS features had nearly three times the odds of CVD compared with women with none, suggesting a cumulative effect potentially mediated by long-term metabolic disturbances.

In order to optimize diagnosis and consequently the choice of a personalized treatment, it can be useful to investigate the presence of potential specific biomarkers which can allow prompt detection of CVD and CMD risk factors in PCOS patients. An interesting perspective described by van der Ham et al. (24) highlights an evolutionary vision of the biomarkers characterizing women with PCOS at major risk of developing cardiometabolic issues during aging. Women with PCOS display unfavorable cardiometabolic biomarkers throughout their lifespan, including BMI, visceral fat, IR, and dyslipidemia, alongside emerging evidence of deregulation in inflammatory cytokines affecting cardiovascular health. While some PCOS characteristics, such as hyperandrogenism, improve with age, cardiometabolic features tend to worsen, forming a complex network of interactions between biomarkers. Screening biomarkers such as tumor necrosis factor α (TNF-α), interleukin 6 (IL-6), monocyte chemoattractant protein-1 (MCP-1), and advanced glycation end-products (AGEs) could aid in assessing cardiovascular risk in women with PCOS, though further research and long-term studies are needed to establish clinical guidelines and reference values.

In addition, a SOMA scan proteomic analysis with BMI matched group between PCOS and controls, revealed an upregulation in several obesity related proteins including: angiopoietin-1 (ANGPT1), Interleukin-1 receptor antagonist protein (IL-1Ra), and lymphotactin, while several cardioprotective proteins resulted downregulated as soluble receptor for advanced glycation end-products (sRAGE), bone morphogenetic protein 6 (BMP6), manganese superoxide dismutase (MnSOD), and growth/differentiation factor 2 (GDF2) (34). An altered expression of these proteins may increase the risk for CVD in overweight/obese patients with PCOS.

In this regard, further in-depth studies and analyses of molecular and genetic associations between PCOS and CVDs could further add important elements to allow an early but also specific diagnosis and to implement effective preventive strategies.

## 5 Different phenotypes also mean different CVD risks

Most of the clinicians refer to the Rotterdam criteria when a diagnosis of PCOS is made. This classification system defines the possible existence of four different subclasses of patients indicated as phenotypes (35). Recent evidence has shed light on the importance of distinguishing between these different phenotypes when PCOS is studied. This crucial point seems highly underestimated to date, and even if increasing data suggest that different phenotypes of PCOS may represent different clinical conditions, these women are frequently considered as a sole group of patients (36).

**Figure 1:**
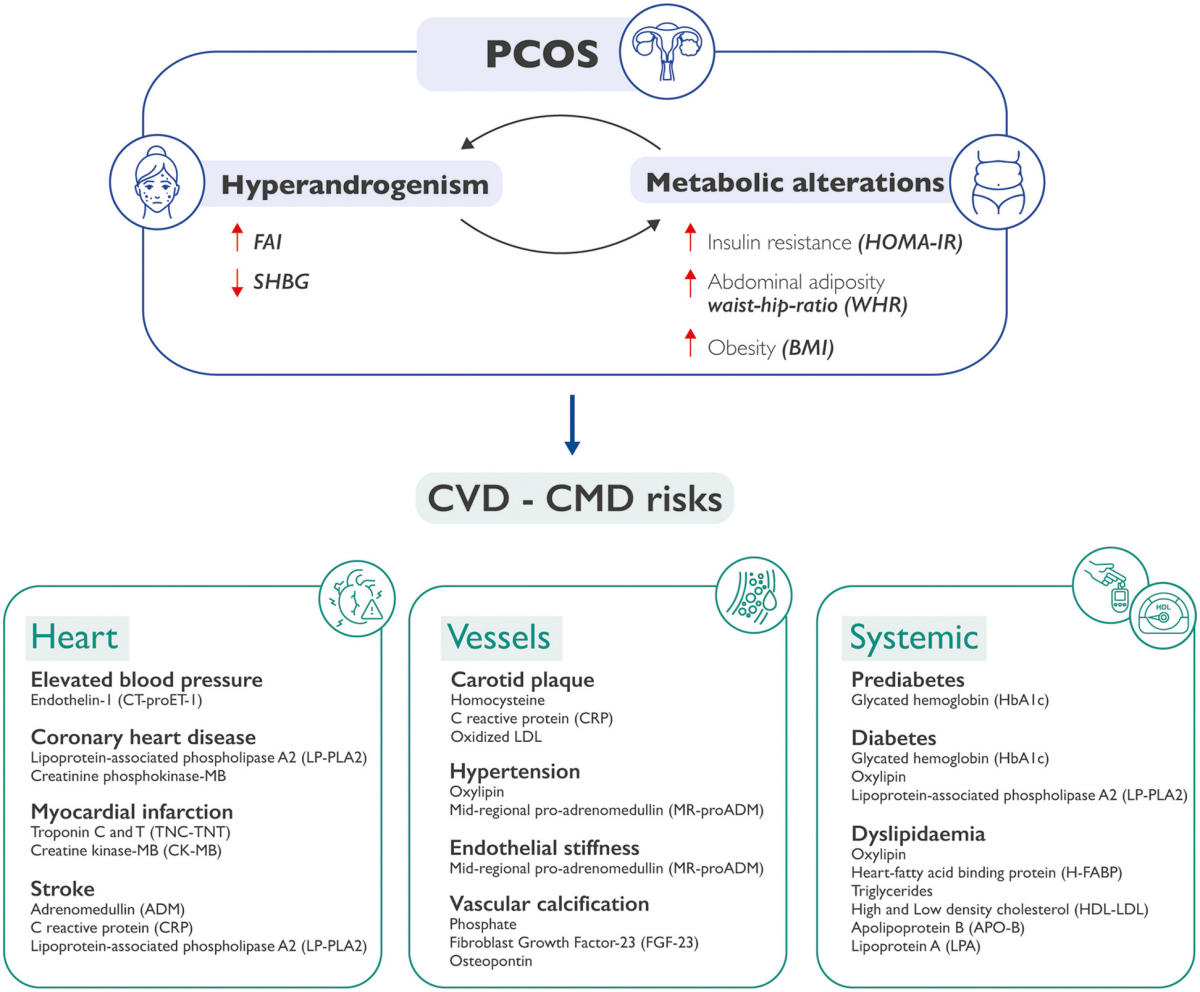
How PCOS may be predictive of CVDs and CMDs. Metabolic alterations can cause hyperandrogenism, and hyperandrogenism in turn can exacerbate metabolic alterations in patients with PCOS. The co-existence of these conditions may lead to an increased cardiovascular and cardiometabolic risk. This could happen at three separate but interrelated levels: heart, vessels, and systemic issues, each one characterized by specific molecular biomarkers. PCOS, polycystic ovary syndrome; CVD, cardiovascular diseases; CMD, cardiometabolic diseases; FAI, free androgen index; SHBG, sex hormone binding globulin; HOMA-IR, Homeostatic Model Assessment of Insulin Resistance; WHR, waist-hip ratio; BMI, body mass index.

Indeed, interesting differences have been observed between those phenotypes that share hyperandrogenism (viz., phenotypes A, B, and C) with respect to the non-hyperandrogenic one (phenotype D). Of note, our group has already published some papers where these differences are accurately investigated to better understand the etiopathogenesis underlying PCOS in its different forms (37). Interestingly, data from literature suggest that CVD risk appears to be more strongly associated with the hyperandrogenic phenotypes which are characterized by an elevated metabolic imbalance. On the other hand, women with phenotype D exhibit a reduced occurrence of metabolic abnormalities and the lowest alteration of insulin sensitivity when compared with the other phenotypes (38). This aspect may reveal an important diagnostic novelty to what is already known in PCOS, uncovering a new pathological axis linking hyperandrogenism–metabolic syndrome-increased CVD risk (39, 40) (Figure 1).

The importance of metabolic alterations and the role of insulin is crucial in defining those women who could be at a higher risk for CVDs and CMDs. Despite deregulation in metabolic features, especially in insulin signaling, which is frequently observed in women with PCOS, these features are not considered or included in most of the proposed classification systems for the syndrome. This is a controversial aspect, considering that it is estimated that approximately 95% of obese women with PCOS and 75% of lean women with PCOS have IR (41, 42).

An interesting prospective study performed in the Finnish population investigated the incidence of CVD in PCOS patients compared with healthy controls, when these patients are classified according to either NIH or Rotterdam criteria (32). Women in the cohort were classified as PCOS at age 31, and they were compared with women without any PCOS features as controls. The study population was re-examined at age 46, and the incidence of major adverse cardiovascular events (MACE), including myocardial infarction (MI), stroke, heart failure, and cardiovascular mortality, was recorded up to age 53. The study concluded that using both classification methods, women with PCOS were significantly more exposed to CVD risks compared with the healthy population. Interestingly, this study also demonstrated that the PCOS group exhibited a significantly higher percentage of metabolic syndrome cases compared with controls.

An interesting finding of this study is that, although the overall rate of metabolic syndrome does not differ significantly between the NIH and Rotterdam classification systems, a clear difference emerges when comparing the incidence of major adverse cardiovascular events (MACE) among PCOS patients. Specifically, women classified using the Rotterdam criteria show a lower risk of MACE compared with those classified under the NIH criteria. This is likely due to a key difference between the two systems: the Rotterdam criteria include non-hyperandrogenic phenotypes (e.g., phenotype D), whereas the NIH definition includes only hyperandrogenic individuals. The inclusion of non-hyperandrogenic women, who are known to have a lower cardiovascular risk, under the Rotterdam system, likely accounts for the observed difference in MACE incidence (43).

These findings underscore the importance of distinguishing between PCOS phenotypes, as their clinical and pathological features vary significantly. In particular, hyperandrogenic PCOS, also referred to in our classification proposal as endocrine–metabolic syndrome (EMS) (44), appears to carry a higher risk of developing cardiovascular disease.

Indeed, the diverse CVD risks may derive from the different metabolic patterns that pertain to each PCOS phenotype. A recent study of Wen et al. (45) involved 442 women with PCOS and described interesting differences between these patients. Women with hyperandrogenic phenotypes had significantly higher BMI, waist circumference, weight–hip ratio, serum insulin, HOMA index (at 2 and 3 h post-glucose), cholesterol, triglycerides, and LDL-C levels than those of women with phenotype D (*p* < 0.05). In addition, the rates of impaired glucose tolerance, IR, metabolic syndrome, nonalcoholic fatty liver disease, and dyslipidemia were significantly higher in the hyperandrogenic group compared with those in the non-hyperandrogenic group (*p* < 0.05). Moreover, elevated testosterone levels (>1.67 nmol/L) and Ferriman–Gallwey scores (>3) significantly increased the risk of metabolic disorders in women with PCOS (*p* < 0.05).

Hyperandrogenism and metabolic disorders may have a mutual impact, potentially creating a self-perpetuating cycle. In hyperandrogenic women, elevated insulin levels, due to impaired insulin function, enhance ovarian androgen production, disrupting the menstrual cycle and promoting features such as hirsutism, acne, and alopecia (46). While peripheral tissues become insulin-resistant, the ovaries remain insulin-sensitive, leading to overactivation of insulin-driven pathways (47). Insulin boosts LH-dependent androgen synthesis and increases D-chiro-inositol, which suppresses aromatase activity and further elevates androgen levels (48). This creates a vicious cycle where hyperandrogenism and insulin resistance reinforce each other.

Thus, further research is needed to explore the mechanisms and precise relationship between hyperandrogenism and metabolic disorders and how this axis directly influences the CDV risk in PCOS patients.

## 6 How to treat these patients, the rationale of oral contraceptive pills (OCPs)

Oral contraceptive pills (OCPs) are common drugs used for PCOS treatment and are composed of ethinyl estradiol combined with a progesterone-like compound. The estrogenic component suppresses LH secretion, reduces the stimulatory input of the ovary, thus inhibiting androgen production. This activity also exerts negative feedback on FSH signaling, thus blocking follicle maturation. Conversely, progestin molecules inhibit the hypothalamic–hypophysis–gonadal axis and turn off the hypothalamic pulse which induces hypothalamic amenorrhea (49).

A report conducted by the Department of Economics and Social Affairs of the United Nations has estimated, with a global survey, that among all the available devices and approaches for contraceptive purposes, 151 million women worldwide use oral contraceptive pills (50). To date, OCPs represent not only a useful approach considered by both patients and physicians for contraceptive purposes, but also OCPs are largely considered as the first-line treatment for PCOS patients (51). Considering that data from literature indicate that approximately 10% of women of reproductive age are affected by PCOS, it can be quickly deduced that we are dealing with 1.51 million potential OCP users with PCOS worldwide. The prescription of OCPs, besides the recognized contraceptive activity, is largely considered in PCOS women suffering from oligo-/anovulation, irregular menstrual cycle, and hyperandrogenism (52). Indeed, OCP is a useful approach to reduce the androgen circulating levels and counteract the aesthetic manifestations of hyperandrogenism such as acne, seborrhea, and alopecia (53). Even though reducing clinical signs of hyperandrogenism and regulating the androgen levels is of primary importance, most of the patients with PCOS suffer from burdensome metabolic alterations. A recent meta-analysis, in which 1,660 pertinent publications were identified and 36 RCTs were included, demonstrates that OCP is not always the best choice of intervention in dysmetabolic patients with PCOS. Indeed, the use of metformin alone or associated with OCPs is proven to be more effective in reducing triglycerides, insulin, and IR in adult women with PCOS compared with the only OCP treatment (54). An analog observation is also documented in adolescent patients exhibiting PCOS, where the treatment with metformin alone or in combination with OCPs is more effective in reducing BMI and dysglycemia, and in improving cholesterol and LDL levels (55). Interestingly, both meta-analyses concluded that the choice of treatment (with insulin sensitizer or OCP) should be based on the symptoms presented by each patient, given that some biochemical parameters could benefit more from a combined treatment.

Of note, the decision-making of the therapeutic approach to recommend should also evaluate the possible risks of adverse events. Even if the cardiovascular risk is negligible in healthy women of reproductive age, the treatment with OCPs correlates with a minimum risk of increasing the blood pressure to date is considered irrelevant. However, when a woman presents with multiple risk factors, it would be appropriate to evaluate whether the administration of OCPs may increase her risk to a no longer acceptable level. In these cases, particularly when these women are smokers and present other risk factors for myocardial infarction and stroke, the use of OCPs is not recommended (56).

Even though OCPs are a safe and effective therapy, over the years, OCP users have experienced several drawbacks and received some warnings on the potential risks and/or side effects. In detail, OCP use may increase the likelihood of developing future complications, such as CVDs, obesity, high blood pressure, hemorrhagic stroke, increasing oxidative stress, risk of cerebral vein thrombosis, and a potential impairment of peak spinal bone mineral density (BMD) that relates to lifetime fracture risk, especially in adolescent using OCPs (57).

## 7 The genetic basis of CVD in PCOS patients

To date, it is not fully disclosed to what extent it is PCOS itself, or its common comorbidities, to mediate the increased risk for CVD in women suffering from this syndrome. Interestingly, recent evidence collected through large-scale genome-wide association studies, the investigation of the genetic basis of PCOS has been able to associate several loci potentially associated with PCOS. These genomic techniques of investigation, including Mendelian randomization (MR), are a useful tool particularly in cardiometabolic disease as they contribute to discovering possible etiological factors (58). A large-scale study used a linkage disequilibrium score regression analysis, which highlighted a genetic correlation between PCOS and diabetes, lipid levels, fasting insulin, obesity, and coronary heart disease, thus overlapping with results from observational studies. Taken together, the results from these studies also suggest a common genetic structure between the cardiometabolic abnormalities and PCOS (59). A great scientific interest has been attracted to the etiopathogenic risk factors of PCOS, and frequently MR studies may result in a useful investigation tool. In this regard, a review of MR studies described a possible causal role in PCOS for several factors including lower serum sex hormone binding globulin concentrations, elevated testosterone levels, higher IR, obesity, delayed menopause occurrence, depression, and various metabolites (60). Moreover, a recent two-sample bidirectional MR analysis carried out for East Asia and Europe highlighted a causal behavior for these populations for increasing BMI and PCOS occurrence, with each standard deviation of genetically higher BMI being associated with as much as 4.89 (1.46–16.32) times higher odds of PCOS (61).

The same approach with MR also revealed that PCOS may not influence CVD in a direct causal manner. Indeed, the authors pointed out that PCOS was not genetically associated with CMD and found no correlation between the genetic prediction of PCOS with the risk of T2D, CHD, or stroke. They concluded that possibly a genetic predisposition to an increased CVD risk may be due not to PCOS itself, but rather to its comorbidities (59).

PCOS condition is commonly recognized as an endocrine–metabolic syndrome (EMS) frequently presenting with some comorbidities as IR, diabetes, dyslipidemia, obesity, and hypertension (62). Interesting evidence in this regard has been recently published following GWAS studies as those for the group of Dapas et al. (63) in 2020. The authors identified the presence of two separate genetic predispositions in PCOS patients, namely, reproductive group and metabolic group, associated with different susceptible loci. The results from the study suggest that these subtypes are biologically relevant because they appear to have distinct genetic architecture, thus highlighting the centrality of metabolic alteration in PCOS onset and development and the genetic association with the classic PCOS phenotypes.

Analogous considerations are retrieved in the study of Hayes et al. (64) designed to identify susceptibility loci for the NIH phenotype of hyperandrogenism and anovulation, which seem to be more frequently associated with high risk for IR and dysglycemia. The study replicated a GWAS carried out for the Chinese population and pointed out that, also in European cohorts, PCOS patients are characterized by alteration of these genes involved in modulating gonadotropin action and secretion. These findings suggest that gonadotropins may play an aetiologic role in the pathogenesis of PCOS.

Genetic studies have also been conducted to evaluate possible sex differences in the etiology of hypertension and CVD risks, even though it is not fully clear whether sex differences in the risk of CVD in children born to PCOS mothers exist. A sample comprised of children born from women with PCOS aged 2.5–8 years, not separated by sex, revealed higher carotid thickness, aortic pressure, and ventricular diameter compared with controls (65). Also, children of PCOS mothers were characterized by increased triglycerides TG and LDL-C compared with controls.

## 8 How to prevent the occurrence of CVD

The presence of PCOS features or a PCOS diagnosis today could also mean a flag of potential CVD occurrence in the future. In this regard, preventive screening and evaluation of CVDRs, along with the diagnosis of PCOS, may be highly beneficial for the patients, as the correct understanding of the clinical picture presented by the patient may help in a rational choice of an intervention from the physicians. In this scenario, the patient should be correctly guided to a therapeutic route in a networking collaboration between the fields of cardiology and gynecology.

In a recent review of Profili et al. (66), some treatments for PCOS and related CVDs were discussed, such as metformin widely used as an insulin sensitizer, GLP-1 receptor agonist as ligralutide, and empagliflozin. The data collected in the review demonstrate that these approaches are useful to improve anthropometric parameters and body composition, thus possibly modifying the CVDRs. Hence, the authors describe a body weight reduction and an improved glucose tolerance with metformin administration, significant decrease of systolic and diastolic blood pressure, weight reduction, and lowered IR, total cholesterol, triglycerides, and serum markers of endothelial functions. Also, gliflozin administration was associated with a decrease in BMI, waist circumference, basal metabolic rate, and fat mass. Interestingly, they concluded that women with PCOS of reproductive age bring an increased risk of developing major CVRFs, particularly metabolic alterations, but today we lack a benchmark for the treatment of this condition. Particular attention should be paid to differentiating PCOS phenotypes to choose the best therapy, further confirming that when we treat PCOS, we are also taking preventive care of CVD progression. In this regard, an interesting and novel approach to improve a preventive diagnosis derives from the possibility of developing specific analytical algorithms designed for PCOS and CVD risks. A recent study suggested a Framingham risk score (FRS), which represents a way to predict the possibility of developing CVDs when PCOS features are diagnosed (67). The authors adopted a dataset containing data from 1998 to 15,005 people with various years of follow-up. The system is based on a specific algorithm with participants divided according to their gender and several CVD risk factors added to calculate the probabilities. Interestingly, the authors discovered that women with PCOS exhibit a 38% increase in the CVD risks during lifespan for each single unit of increased FRS. The authors further highlight the importance of screening for CVD risks in PCOS with reliable tools to allow a prompt and complete management of women with PCOS.

Quite often, OCPs are considered a golden standard for PCOS treatment considering that the treatment with OCPs may lower androgen levels in PCOS patients, thus balancing the endocrine alterations (68). Of note, even if OCPs may result in a useful tool to reduce androgens and the aesthetic manifestation of hyperandrogenism, the effect that could be observed on the metabolic profile is merely indirect. Moreover, as aforementioned, patients with PCOS might carry a genetic predisposition to IR and altered glucose metabolism that could not be rescued with OCPs intervention. Differently, an insulin-sensitizing therapy, particularly in adolescence, may help the “education” of the ovary, thus preventing both dysmetabolism occurrence and hyperandrogenism derived from excessive insulin stimulus of the ovarian theca cells.

## 9 Conclusions

Reducing the CVD burden among women remains challenging. Epidemiologic studies have indicated that PCOS, the most common endocrine disease in women of reproductive age, is associated with an increased prevalence and extent of coronary artery disease. It seems clear that PCOS is strongly associated with increased CVD risk, but it becomes fundamental to distinguish between the different phenotypes of PCOS to better address the risk grade. Most of the patients with PCOS share hyperandrogenism both in its biochemical and clinical form, and the same patients seem more predisposed to develop metabolic alterations, also considering their genetic structure. In case of PCOS, particularly for hyperandrogenic women, physicians frequently recommend the OCPs to reduce androgen levels and restore menstrual regularity, but quite often this intervention is not effective in reducing the CVD risk in these patients. Hence, the OCP treatment does not seem to be effective on the metabolic alteration where the increased risk of CVD effectively lies. A correct diagnosis of PCOS becomes fundamental for the prevention of CVDs occurrence in the future and also to facilitate the treatment choice depending on the subtype of patient presenting with PCOS. Considering the risk of taking OCP, if prescribed in patients with PCOS, careful 6-monthly monitoring is strictly recommended.
